# Serum levels of vascular endothelial growth factor are increased and correlate with malnutrition, immunosuppression involving MDSCs and systemic inflammation in patients with cancer of the digestive system

**DOI:** 10.3892/ol.2013.1231

**Published:** 2013-03-06

**Authors:** IZUMI NAKAMURA, MASAHIKO SHIBATA, KENJI GONDA, TAKASHI YAZAWA, TATSUO SHIMURA, TAKAYUKI ANAZAWA, SATOSHI SUZUKI, KENICHI SAKURAI, YOSHIHISA KOYAMA, HITOSHI OHTO, RYOUICHI TOMITA, MITSUKAZU GOTOH, SEIICHI TAKENOSHITA

**Affiliations:** 1 Departments of Organ Regulatory Surgery, Fukushima 960-1295;; 2 Tumor and Host Bioscience, Fukushima 960-1295;; 3 Blood Transfusion and Transplantation Immunology, Fukushima 960-1295;; 4 Regenerative Surgery, Fukushima Medical University, Fukushima 960-1295;; 5 Department of Surgery, Nihon University School of Medicine, Itabashi, Tokyo 173-8610;; 6 Department of Surgery, Nippon Dental University, Chiyoda, Tokyo 102-8158, Japan

**Keywords:** vascular endothelial growth factor, myeloid-derived suppressor cells, immunosuppression, systemic inflammation, cachexia, gastric cancer, colorectal cancer

## Abstract

Vascular endothelial growth factor (VEGF) reportedly has an important role in the progression of malignant neoplasms and has been reported to induce myeloid-derived suppressor cells (MDSCs) that appear in cancer and inflammation. In the present study, serum concentrations of VEGF were measured in patients with digestive system cancer and the correlations with nutritional damage, immune suppression and systemic inflammation were analyzed. A significant increase in VEGF serum levels was observed in patients with esophageal, gastric and colorectal cancers compared with healthy volunteers. Levels of VEGF were inversely correlated with the serum concentrations of albumin, prealbumin and retinol-binding protein. The serum concentrations of VEGF were inversely correlated with the production of interleukin (IL)-12 and correlated with MDSC counts. VEGF levels were also correlated with neutrophil and neutrophil/lymphocyte counts and inversely correlated with lymphocyte count. Serum VEGF levels were divided at a cutoff of 500 pg/ml, with levels of prealbumin and retinol-binding protein significantly decreased in patients with higher VEGF levels. The stimulation index and IL-12 production were significantly decreased in the group with higher VEGF levels and MDSC counts tended to be higher in this group. These results demonstrated that increased production of VEGF was correlated with systemic inflammation, nutritional impairment and the inhibition of cell-mediated immunity involving MDSCs.

## Introduction

The observation that angiogenesis occurs around neoplastic tumors was made more than a century ago (1). Tumor growth and metastasis were subsequently proposed to depend on angiogenesis and the blockage of angiogenesis may thus provide one strategy for inhibiting tumor growth. The involvement of angiogenesis and vascular endothelial growth factor (VEGF) in the pathogenesis of neoplastic diseases has been described (2).

VEGF, previously known as vascular permeability factor, has a mass of 45 kDa and belongs to a family of platelet-derived growth factors. Thus far, several forms of VEGF have been distinguished, including isoforms A, B, C, D and E (3,4). The biological significance of the different forms of VEGF has yet to be definitively recognized.

Elevated VEGF levels are reportedly associated with advanced-stage melanoma, along with negative immune reactions, including type 2 helper T cell (Th2) dominance and impaired dendritic cell function (5). Studies have identified myeloid-origin cells that are potent suppressors of tumor immunity and therefore represent a significant impediment to cancer immunotherapy. Suppressive myeloid cells were described three decades ago in patients with cancer, but their functional importance in the immune system has only recently been shown (6). Accumulating evidence is now showing that myeloid-derived suppressor cells (MDSCs) contribute to the negative regulation of immune responses in cancer and other diseases. In contrast to the situation in mice, the pathophysiological functions of MDSCs have not been clarified as well in patients. However, induction and expansion of the MDSC population is associated with cancer and inflammation (7).

Cachexia due to cancer is a complex metabolic disorder, with symptoms including loss of adipose tissue due to lipolysis, loss of skeletal muscle, elevation of resting energy consumption, anorexia and reduction of oral food intake (8). Acute-phase response proteins including VEGF have been reported to be associated with the development of cachexia in patients with cancer (9). The present study investigated the status of VEGF and examined associations between serum levels of VEGF and markers of nutrition and inflammation, as well as levels of MDSCs, in patients with cancer of the digestive system.

## Materials and methods

### Sample collection

Blood samples were collected from 100 patients, including 8 with esophageal cancer, 20 with gastric cancer, 29 with colorectal cancer, 20 with hepatocellular carcinoma, 12 with cholangiocellular carcinoma and 11 with pancreatic carcinoma, as well as 18 healthy volunteers with similar age and gender distributions. The esophageal cancer patient group included 2 patients with stage II disease, 2 with stage III and 4 with stage IV, while the 20 patients with gastric cancer included 7 with stage I, 4 with stage II, 3 with stage III and 6 with stage IV disease. The 29 patients with colorectal cancer included 3 with stage I, 10 with stage II, 8 with stage III and 8 with stage IV disease. The 20 patients with hepatocellular carcinoma included 4 with stage I, 6 with stage II, 2 with stage III and 8 with stage IV disease. The 12 patients with cholangiocellular carcinoma included 3 with stage I, 1 with stage II, 3 with stage III and 5 with stage IV disease. The 11 patients with pancreatic cancer included 1 with stage I, 1 with stage II, 3 with stage III and 6 with stage IV disease. All enrolled patients had received surgery or chemotherapy in the Department of Organ Regulatory Surgery or the Department of Regenerative Surgery at Fukushima Medical University Hospital between January 2011 to May 2012 and were 34–94 years old (median, 65.1 years) with histological confirmation of the diagnosis. All patients were newly diagnosed and blood samples were collected before any treatment was initiated.

The present study was approved by the ethics committee at Fukushima Medical University (2010-204) and written informed consent was obtained from all subjects.

### Isolation of peripheral blood mononuclear cells (PBMCs)

PBMCs were isolated with Ficoll-Hypaque (Pharmacia-Biotech, Uppsala, Sweden), washed twice with RPMI-1640 (Wako Pure Chemical Industries, Osaka, Japan) and stored in freezing medium (BLC-1; Juji-Field, Tokyo, Japan) at −80°C until use, all according to standard methods.

### Serum levels of VEGF

Peripheral venous blood sera from all subjects were stored at −80°C until use. Serum concentrations of VEGF were measured using enzyme-linked immunosorbent assays (ELISAs; R&D Systems, Minneapolis, MN, USA) in accordance with the manufacturer’s instructions.

### Flow cytometry

**Figure 1:**
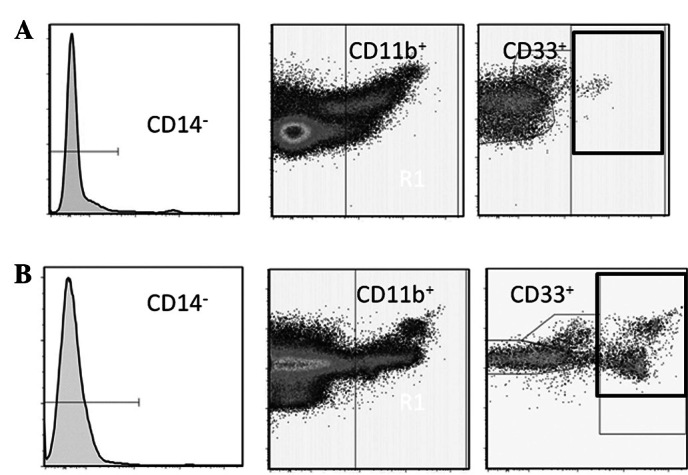
Immunophenotyping of MDSCs by flow cytometry. Cells were labeled with FITC, PE and PC5. Antibodies included those targeting FITC-conjugated CD14, PE-conjugated CD11b and PC5-conjugated CD33. (A) Healthy volunteers; (B) colon cancer patients. MDSC, myeloid-derived suppressor cells; FITC, fluorescent isothiocyanate; PE, phycoerythrin; PC5, phycoerythrin cyanin 5.1.

Cells were immunofluorescence-labeled and analyzed by flow cytometry. The labels used included fluorescent isothiocyanate (FITC), phycoerythrin (PE) and PE cyanin 5.1 (PC5; Beckman Coulter, Marseille, France). The antibodies were used at 10 and 50 *μ*g/ml and were diluted in phosphate-buffered saline (PBS). Cells were incubated with antibodies for 20 min at 4°C, then washed with PBS. The antibodies used included FITC-conjugated CD14 (Abcam, Cambridge, UK), PE-conjugated CD11b (Beckman Coulter) and PC5-conjugated CD33 (Beckman Coulter). Data acquisition and analysis were performed on a FACS Aria II flow cytometer (BD Biosciences, Mountain View, CA, USA) using Flow Jo software (Tree Star, Inc., Ashland, OR, USA). The typical pattern of analysis is shown in Fig. 1.

### Lymphocyte proliferation assay

Lymphocyte proliferation assays were performed using PBMCs suspended in RPMI-1640 (Wako Pure Chemical Industries) containing 10% fetal calf serum (Sigma, St. Louis, MO, USA). After the addition of 10 *μ*g/ml phytohemagglutinin (PHA) into PBMC culture wells stored at 37°C in a 5% CO_2_ atmosphere, PHA mitogenesis was observed for 80 h. ^3^H-thymidine (Japan Radioisotope Association, Tokyo, Japan) was added to wells for the last 8 h of incubation. Cells were harvested and ^3^H-thymidine incorporation was determined using a liquid scintillation counter (Perkin-Elmer, Waltham, MA, USA) and expressed as counts per minute (cpm). The stimulation index (SI) was obtained by calculating total cpm / control cpm. The controls were PBMCs that had not been subjected to PHA addition.

### Cytokine production by PBMCs

Samples (20 ml) of blood obtained directly from heparinized collection tubes were subjected to Ficoll density gradient centrifugation to isolate the PBMCs, 10^6^ of which were incubated in 1 ml of RPMI-1640 medium containing 10% heat-inactivated fetal calf serum (Gibco BRL, St. Louis, MO, USA) and 20 *μ*g/ml PHA (Sigma, Rockville, MD, USA) in 5% CO_2_ at 37°C for 24 h. Aliquots of these supernatants were then frozen and stored at −80°C until use. Supernatant samples were subsequently thawed and used for measurement of IL-12 concentrations using ELISA kits (R&D Systems). Each sample was used only once after thawing.

### Markers for nutritional status and chronic inflammation

The patients’ nutritional status was determined by measuring serum concentrations of albumin (nephelometry), prealbumin (turbidimetric immunoassay), retinol-binding protein (latex agglutination immunoassay) and transferrin (turbidimetric immunoassay). Neutrophil and lymphocyte counts, as well as ratios such as the neutrophil/lymphocyte ratio (NLR) in peripheral blood samples, were used as indicators of inflammation.

### Statistical analysis

Differences between groups were determined using the Student’s t-test. Associations between two variables were quantified using the Spearman’s rank correlation coefficient. P<0.05 was considered to indicate statistically significant differences. Not all blood samples were of sufficient volume for all measurements.

## Results

**Figure 2:**
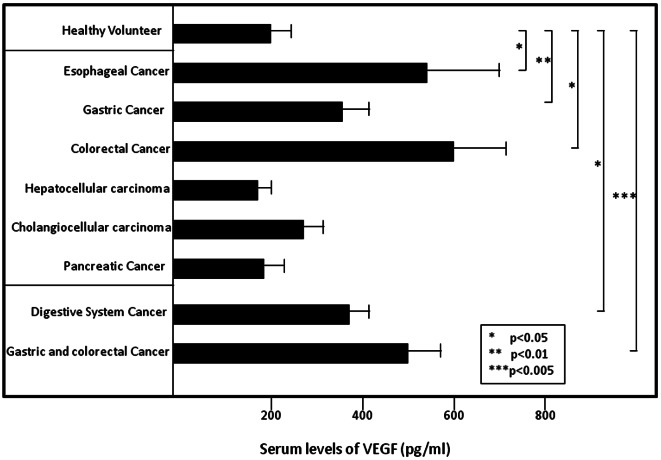
Serum VEGF concentrations in patients with digestive system cancer. Significant increases in serum levels were observed in patients with eso phageal (573.0±150.6 pg/ml, P<0.01), gastric (360.8±56.9 pg/ml, P<0.01), colo rectal (601.8±128.3 pg/ml, P<0.05), whole digestive cancer (369.2±44.9 pg/ml, P<0.05) and gastric and colorectal cancer (499.4±79.6 pg/ml, P<0.005) compared with healthy volunteers (217.8±46.3 pg/ml). VEGF, vascular endothelial growth factor.

**Figure 3:**
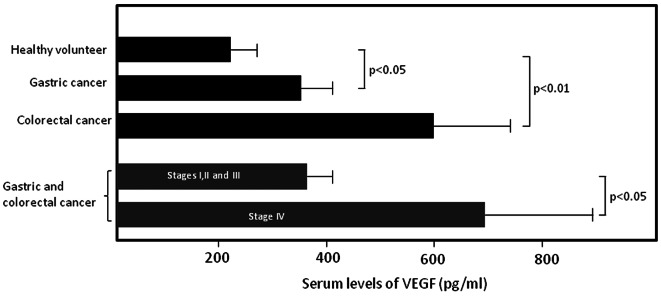
Serum VEGF concentrations in patients with gastric and colorectal cancer. The mean serum VEGF level was 365.2±43.0 pg/ml in patients with stage I, II and III cancer and 695.1±192.7 pg/ml in patients with stage IV cancer. These levels were significantly elevated compared with healthy volunteers (P<0.05 and P<0.01, respectively) and the stage IV VEGF levels were also significantly increased compared with those of the other stages (P<0.05). VEGF, vasuclar endothelial growth factor.

**Figure 4:**
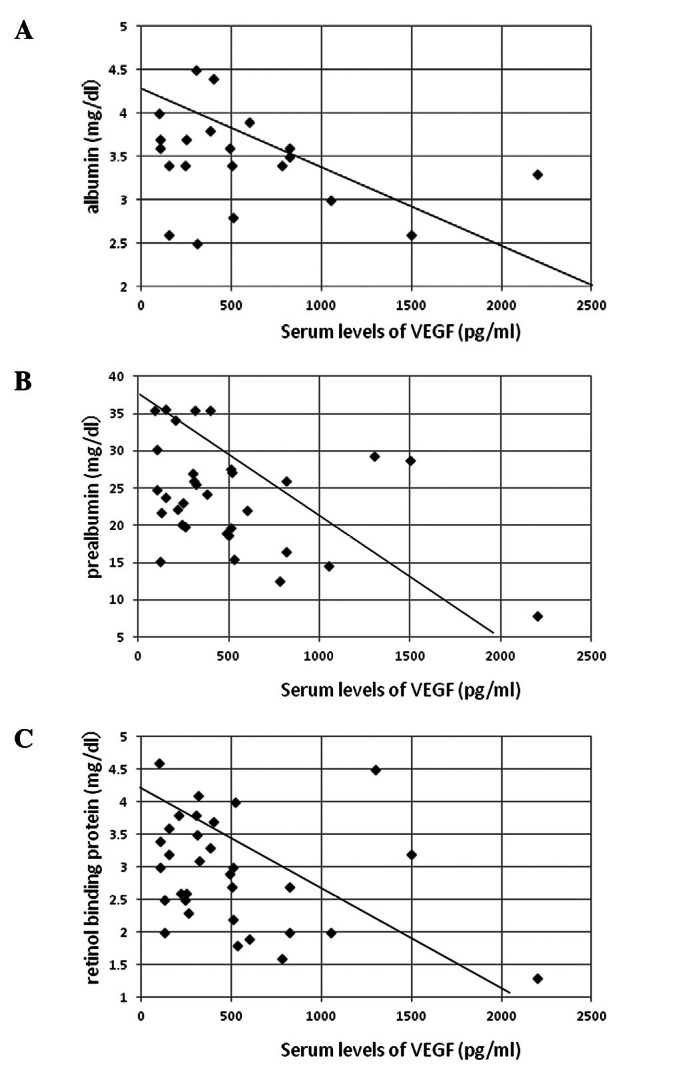
Correlation of serum VEGF levels of with nutritional markers in gastric and colorectal cancer. Levels of VEGF showed significant inverse correlations with the serum concentrations of (A) albumin (r=−0.447, P<0.05), (B) prealbumin (r=−0.465, P<0.001) and (C) retinol-binding protein (r=−0.408, P<0.05). VEGF, vasuclar endothelial growth factor.

**Figure 5:**
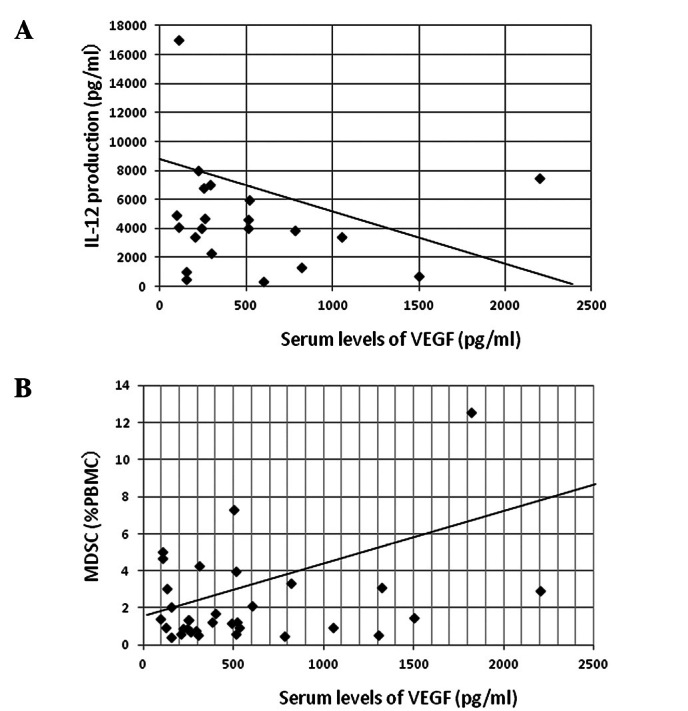
Correlation of serum VEGF levels with immune reaction in gastric and colorectal cancer. Serum concentrations of VEGF correlated inversely with (A) production of IL-12 (r=−0.659, P<0.05) and (B) MDSC counts (r= 0.409, P<0.01). VEGF, vascular endothelial growth factor; IL-12, interleukin 12; MDSC, myeloid-derived suppressor cells.

**Figure 6:**
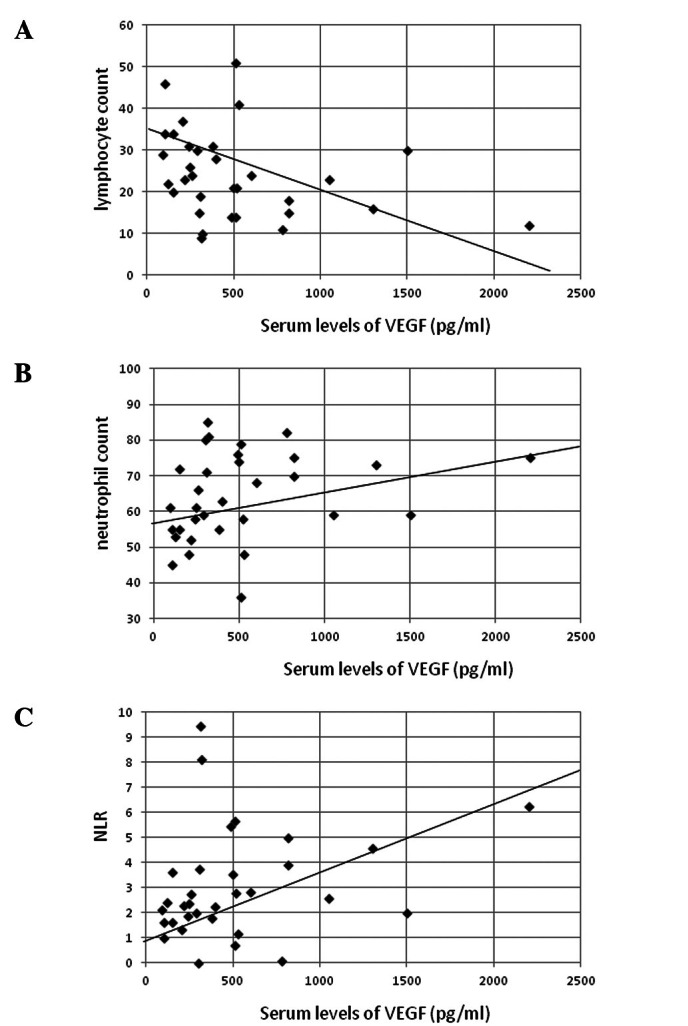
Correlation of serum VEGF levels with markers of inflammation in gastric and colorectal cancer. Serum concentrations of VEGF were also correlated with (A) neutrophil count (r= 0.297, P<0.05) and (B) NLR (r= 0.277, P<0.05) and inversely correlated with (C) lymphocyte count (r=−0.341, P<0.05). VEGF, vascular endothelial growth factor; NLR, neutrophil/lymphocyte ratio.

PBMCs obtained from 100 patients with various types of digestive system cancer and 18 normal volunteers were tested. Significant increases were observed in serum VEGF levels for patients with esophageal (573.0±150.6 pg/ml, P<0.01), gastric (360.8±56.9 pg/ml, P<0.01), colorectal (601.8±128.3 pg/ml, P<0.05), whole digestive (369.2±44.9 pg/ml, P<0.05) and gastric and colorectal cancer (499.4±79.6 pg/ml, P<0.005) compared with healthy volunteers (217.8±46.3 pg/ml; Fig. 2). Patients with hepatocellular (173.0±29.4 pg/ml), cholangio-cellular (271.6±85.6 pg/ml, P<0.05) and pancreatic carcinoma (191.0±44.6 pg/ml) did not show significant differences from the healthy volunteers. The following analyses were performed in patients with gastric and colorectal cancer. The serum level of VEGF in patients with stage I–III cancer was 365.2±43.0, compared with 695.1±192.7 pg/ml for stage IV. These levels were significantly elevated compared with those of healthy volunteers (P<0.05 and P<0.01, respectively) and the stage IV VEGF levels were also significantly increased compared with those of the other stages (P<0.05; Fig. 3). These data were analyzed for correlations with nutritional status, immune function and inflammation. VEGF levels exhibited significant inverse correlations with the serum concentrations of albumin (r=−0.447, P<0.05), prealbumin (r=−0.465, P<0.001) and retinol-binding protein (r=−0.408, P<0.05; Fig. 4). An inverse correlation was observed with the production of IL-12 (r=−0.659, P<0.005) and a significant correlation was observed for MDSC counts (r= 0.409, P<0.01; Fig. 5). Correlations of VEGF levels were also observed with the neutrophil count (r= 0.297, P<0.05) and NLR (r= 0.277, P<0.05), as well as an inverse correlation with the lymphocyte count (r=−0.341, P<0.05; Fig. 6). In addition, dividing the VEGF levels at a cutoff of 500 pg/ml showed that the levels of prealbumin and retinol-binding protein were significantly decreased in patients with higher VEGF levels (P<0.05, both) and similar tendencies were observed for albumin and transferrin (P<0.10 for both, Table I). The SI, determined by assessing lymphocyte PHA-blastogenesis, and IL-12 production were significantly decreased at higher VEGF levels (P<0.05 and P<0.01, respectively), while MDSC counts tended to be higher in this group (P<0.10).

## Discussion

VEGF has been reported to have an important role in the progression of malignant neoplasms (10). In the present study, the associations of VEGF with nutritional damage, immune suppression and systemic inflammation were investigated. The VEGF level was significantly higher in patients with certain types of gastrointestinal cancer compared with normal volunteers and was higher still in patients with stage IV gastric or colorectal cancer. Significant correlations were observed with neutrophil count and NLR. By contrast, VEGF levels showed significant inverse correlations with nutritional condition, as reflected by albumin, prealbumin and retinol-binding protein, and cell-mediated immune responses in Th1 induction, as reflected by IL-12 production. The circulating MDSC counts were also significantly correlated with VEGF levels. In addition, higher levels of malnutrition and immune suppression were observed in patients with higher VEGF levels. These results clearly support the hypothesis that VEGF is important for immune suppression, malnutrition and inflammation, which are essential factors for the progression of digestive system cancer, indicating the possible involvement of VEGF in the pathogenesis of cachexia.

Decreased albumin concentrations are involved with cachexia and are common laboratory features in malignant diseases. Hypoalbuminemia has been demonstrated to be a predictive factor for poor responsiveness (11,12). The ongoing systemic inflammatory response in terms of NLR has received interest, as an easily measured and standardized predictor of outcomes in patients following treatment (13,14).

The immunosuppressive properties of malignant tumors have been reported previously. Central to this concept is the polarization of the immune system toward a state of inflammation driven by immunological mediators produced by tumor and immune cells (15,16). NLR has been reported to be a marker for systemic inflammatory responses and an independent predictor of clinical benefit, good prognosis and survival in patients receiving cancer chemotherapy. We have previously reported that MDSCs are increased in numerous types of cancer (17,18) and the present study observed a significant correlation between VEGF levels and the number of circulating MDSCs. The exact mechanisms involved in the increased production of immature myeloid cells in cancer patients remain unclear. However, tumor cells are known to produce several growth factors and cytokines that are able to stimulate myelopoiesis (19,20). One possibility is that increased production of these growth factors may affect normal cell differentiation, resulting in the accumulation of immature myeloid cells. In addition, VEGF has been shown to induce MDSC production, which suppresses dendritic cell function (6,21). This linkage may result in decreased IL-12 production by dendritic cells affected by VEGF and lead to the decrease in lymphocyte blastogenesis observed in the present study.

The present results demonstrated that increased production of VEGF is correlated with systemic inflammation, nutritional impairment and inhibition of cell-mediated immunity, with the involvement of MDSCs. Future studies should investigate the possibilities for clinical control of immune suppression and chronic inflammation by modulating VEGF.

## Figures and Tables

**Table I:** Comparison of patients with high and low serum levels of VEGF.

Factor	Patients with high VEGF (n=13)	Patients with low VEGF (n=20)	P-value
Albumin (mg/dl)	3.28±0.13	3.60±0.17	<0.10
Transferrin (mg/dl)	240.66±13.10	253.82±11.32	<0.10
Prealbumin (mg/dl)	20.44±1.92	26.22±1.47	<0.05
Retinol binding protein (mg/dl)	2.53±0.26	3.18±0.15	<0.05
Stimulation index	526.14±66.90	750.71±76.50	<0.05
IL-12 production (pg/ml)	3078.26±636.45	5750.03±1280.22	<0.01
MDSC (% PBMC)	2.97±0.95	2.27±0.52	<0.10

Patients were categorized according to VEGF levels at a cutoff of 500 pg/ml. Levels of prealbumin and retinol-binding protein were significantly decreased in patients with higher VEGF levels (P<0.05, both). Similar tendencies were evident for albumin and transferrin (P<0.10, both). In patients with higher VEGF levels, SI (determined by assessing lymphocyte PHA-blastogenesis) and IL-12 production were significantly decreased (P<0.05 and P<0.01, respectively) and MDSC counts tended to be higher (P<0.10). VEGF, vascular endothelial growth factor; MDSC, myeloid-derived suppressor cells; SI, stimulation index; PHA, phytohemagglutinin; IL-12, interleukin 12.
